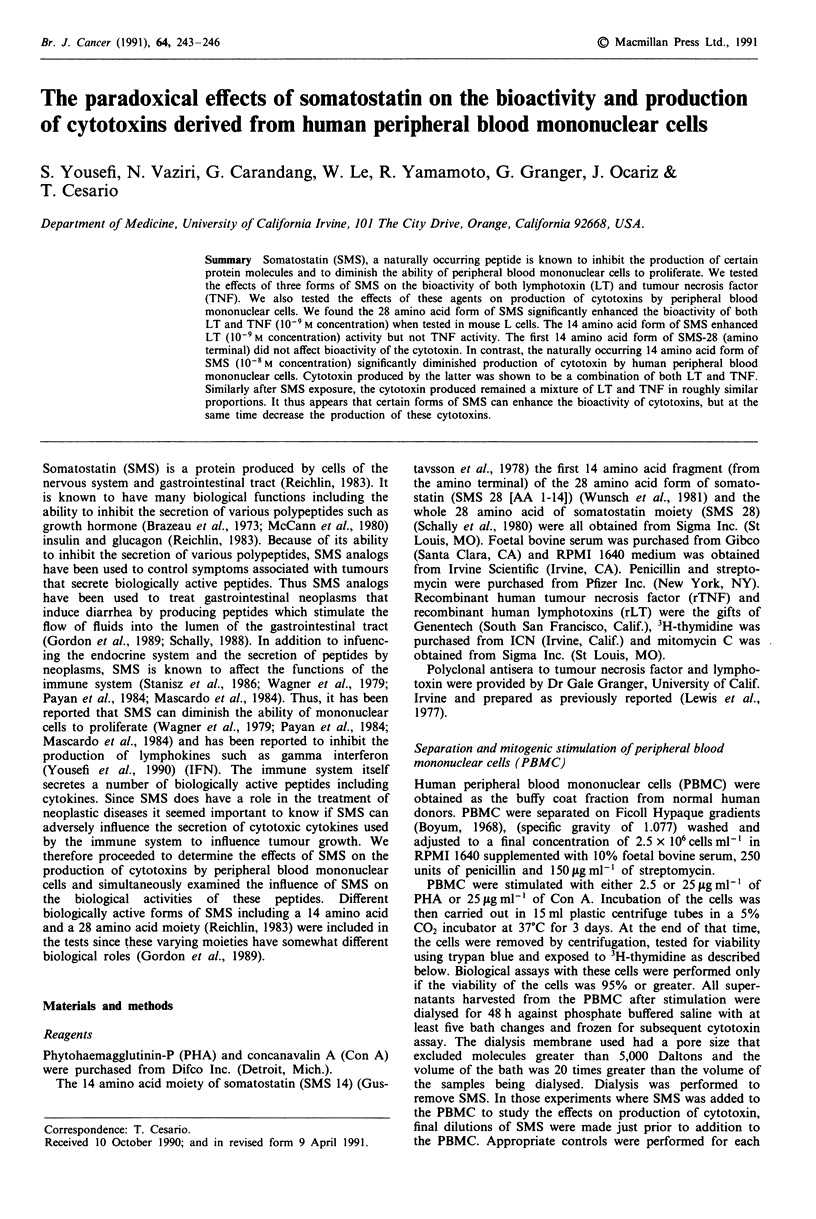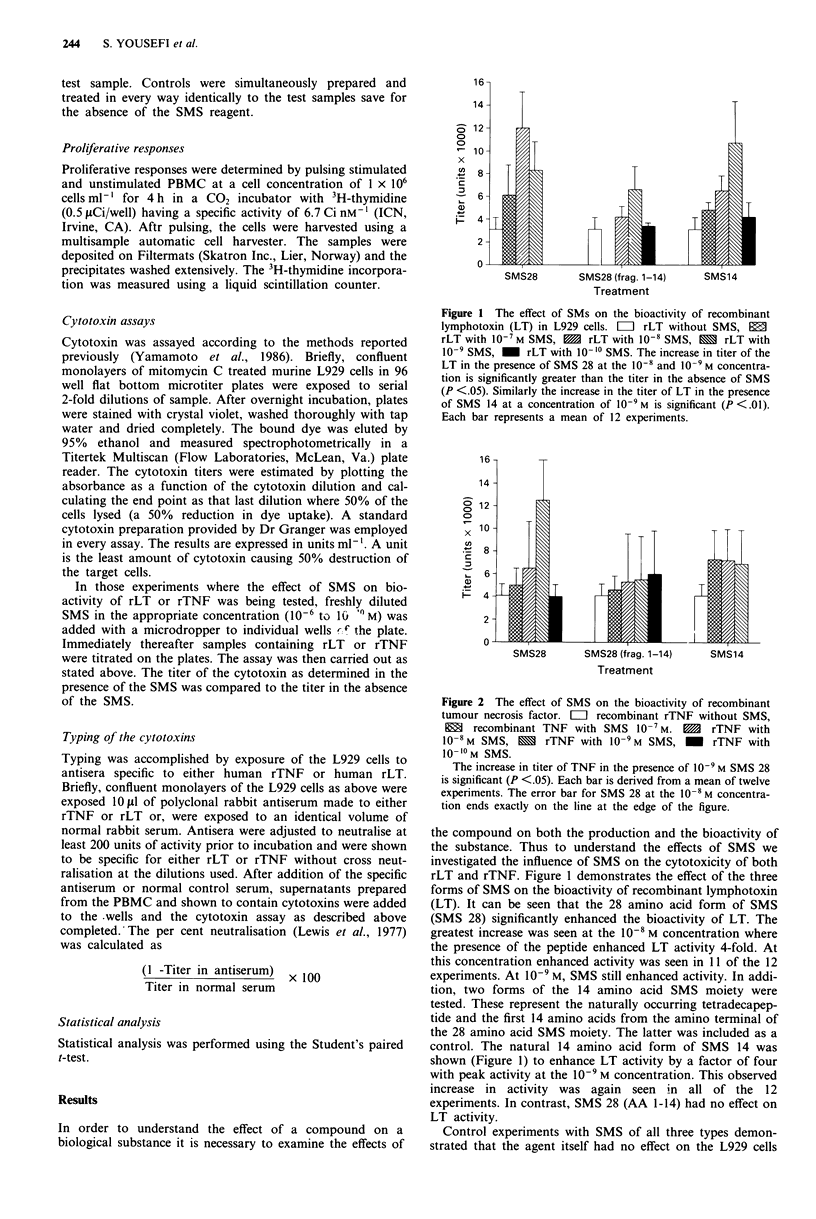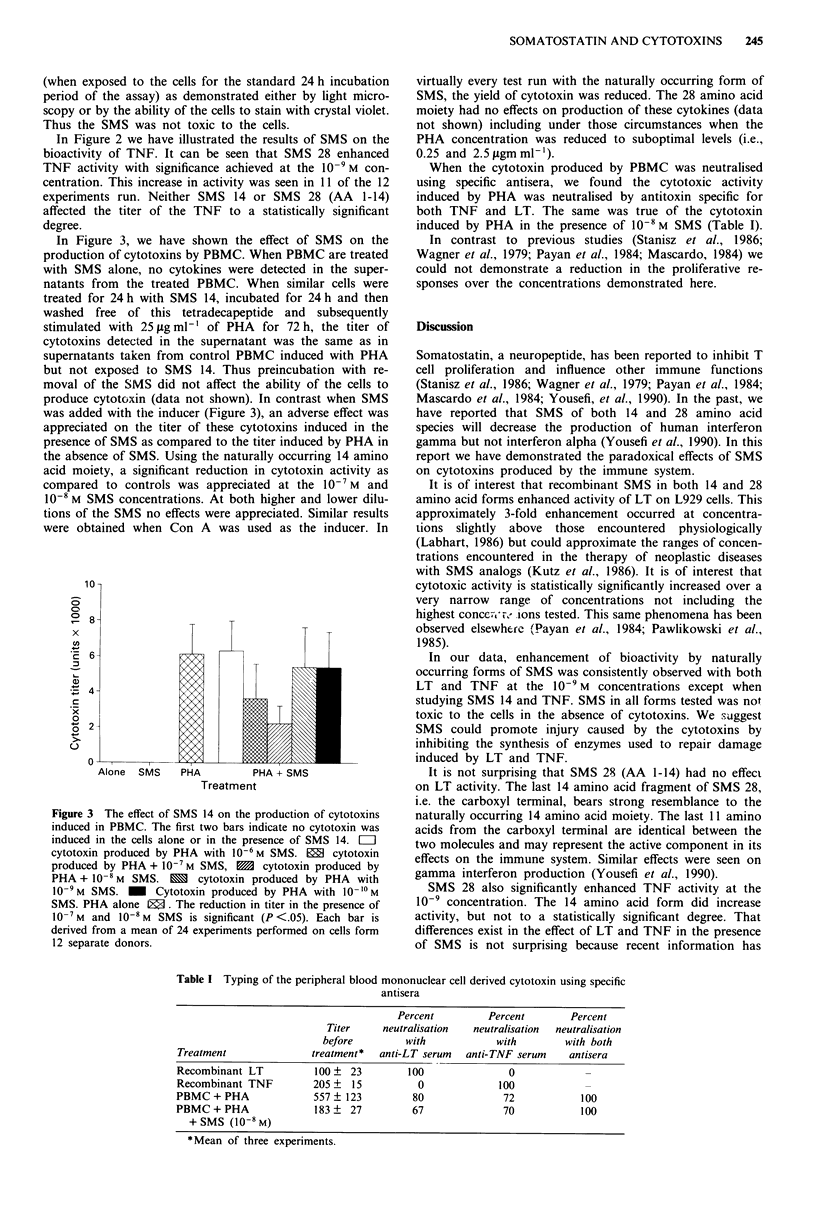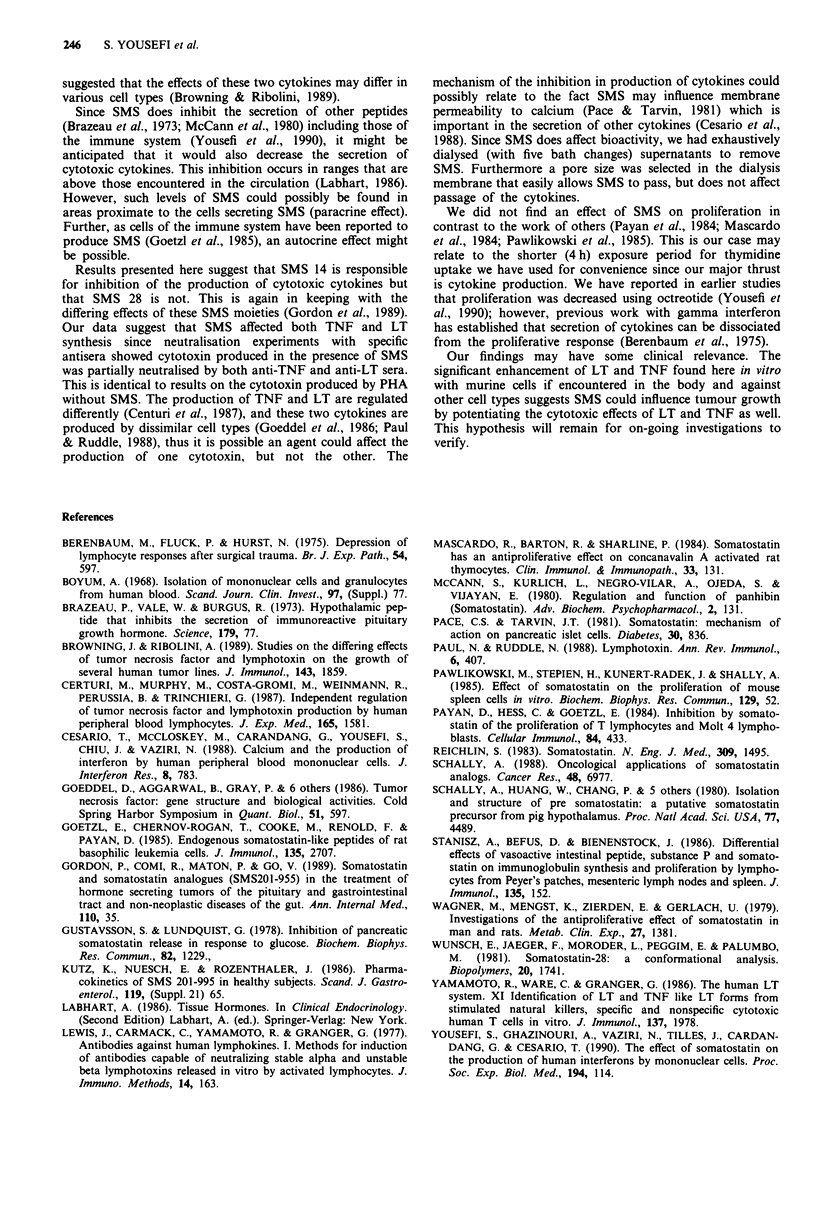# The paradoxical effects of somatostatin on the bioactivity and production of cytotoxins derived from human peripheral blood mononuclear cells.

**DOI:** 10.1038/bjc.1991.284

**Published:** 1991-08

**Authors:** S. Yousefi, N. Vaziri, G. Carandang, W. Le, R. Yamamoto, G. Granger, J. Ocariz, T. Cesario

**Affiliations:** Department of Medicine, University of California Irvine, Orange 92668.

## Abstract

Somatostatin (SMS), a naturally occurring peptide is known to inhibit the production of certain protein molecules and to diminish the ability of peripheral blood mononuclear cells to proliferate. We tested the effects of three forms of SMS on the bioactivity of both lymphotoxin (LT) and tumour necrosis factor (TNF). We also tested the effects of these agents on production of cytotoxins by peripheral blood mononuclear cells. We found the 28 amino acid form of SMS significantly enhanced the bioactivity of both LT and TNF (10(-9) M concentration) when tested in mouse L cells. The 14 amino acid form of SMS enhanced LT (10(-9) M concentration) activity but not TNF activity. The first 14 amino acid form of SMS-28 (amino terminal) did not affect bioactivity of the cytotoxin. In contrast, the naturally occurring 14 amino acid form of SMS (10(-8) M concentration) significantly diminished production of cytotoxin by human peripheral blood mononuclear cells. Cytotoxin produced by the latter was shown to be a combination of both LT and TNF. Similarly after SMS exposure, the cytotoxin produced remained a mixture of LT and TNF in roughly similar proportions. It thus appears that certain forms of SMS can enhance the bioactivity of cytotoxins, but at the same time decrease the production of these cytotoxins.


Br. J. Cancer (1991), 64, 243-246 © Macmillan Press Ltd., 1991

### The paradoxical effects of somatostatin on the bioactivity and production of cytotoxins derived from human peripheral blood mononuclear cells

S. Yousefi, N. Vaziri, G. Carandang, W. Le, R. Yamamoto, G. Granger, J. Ocariz & T. Cesario

*Department of Medicine, University of California Irvine, 101 The City Drive, Orange, California 92668, USA.*

**Summary** Somatostatin (SMS), a naturally occurring peptide is known to inhibit the production of certain protein molecules and to diminish the ability of peripheral blood mononuclear cells to proliferate. We tested the effects of three forms of SMS on the bioactivity of both lymphotoxin (LT) and tumour necrosis factor (TNF). We also tested the effects of these agents on production of cytotoxins by peripheral blood mononuclear cells. We found the 28 amino acid form of SMS significantly enhanced the bioactivity of both LT and TNF ($10^{-9}$ M concentration) when tested in mouse L cells. The 14 amino acid form of SMS enhanced LT ($10^{-9}$ M concentration) activity but not TNF activity. The first 14 amino acid form of SMS-28 (amino terminal) did not affect bioactivity of the cytotoxin. In contrast, the naturally occurring 14 amino acid form of SMS ($10^{-8}$ M concentration) significantly diminished production of cytotoxin by human peripheral blood mononuclear cells. Cytotoxin produced by the latter was shown to be a combination of both LT and TNF. Similarly after SMS exposure, the cytotoxin produced remained a mixture of LT and TNF in roughly similar proportions. It thus appears that certain forms of SMS can enhance the bioactivity of cytotoxins, but at the same time decrease the production of these cytotoxins.

Somatostatin (SMS) is a protein produced by cells of the nervous system and gastrointestinal tract (Reichlin, 1983). It is known to have many biological functions including the ability to inhibit the secretion of various polypeptides such as growth hormone (Brazeau *et al.*, 1973; McCann *et al.*, 1980) insulin and glucagon (Reichlin, 1983). Because of its ability to inhibit the secretion of various polypeptides, SMS analogs have been used to control symptoms associated with tumours that secrete biologically active peptides. Thus SMS analogs have been used to treat gastrointestinal neoplasms that induce diarrhea by producing peptides which stimulate the flow of fluids into the lumen of the gastrointestinal tract (Gordon *et al.*, 1989; Schally, 1988). In addition to infuencing the endocrine system and the secretion of peptides by neoplasms, SMS is known to affect the functions of the immune system (Stanisz *et al.*, 1986; Wagner *et al.*, 1979; Payan *et al.*, 1984; Mascardo *et al.*, 1984). Thus, it has been reported that SMS can diminish the ability of mononuclear cells to proliferate (Wagner *et al.*, 1979; Payan *et al.*, 1984; Mascardo *et al.*, 1984) and has been reported to inhibit the production of lymphokines such as gamma interferon (Yousefi *et al.*, 1990) (IFN). The immune system itself secretes a number of biologically active peptides including cytokines. Since SMS does have a role in the treatment of neoplastic diseases it seemed important to know if SMS can adversely influence the secretion of cytotoxic cytokines used by the immune system to influence tumour growth. We therefore proceeded to determine the effects of SMS on the production of cytotoxins by peripheral blood mononuclear cells and simultaneously examined the influence of SMS on the biological activities of these peptides. Different biologically active forms of SMS including a 14 amino acid and a 28 amino acid moiety (Reichlin, 1983) were included in the tests since these varying moieties have somewhat different biological roles (Gordon *et al.*, 1989).

### Materials and methods

### *Reagents*

Phytohaemagglutinin-P (PHA) and concanavalin A (Con A) were purchased from Difco Inc. (Detroit, Mich.).

The 14 amino acid moiety of somatostatin (SMS 14) (Gustavsson *et al.*, 1978) the first 14 amino acid fragment (from the amino terminal) of the 28 amino acid form of somatostatin (SMS 28 [AA 1-14]) (Wunsch *et al.*, 1981) and the whole 28 amino acid of somatostatin moiety (SMS 28) (Schally *et al.*, 1980) were all obtained from Sigma Inc. (St Louis, MO). Foetal bovine serum was purchased from Gibco (Santa Clara, CA) and RPMI 1640 medium was obtained from Irvine Scientific (Irvine, CA). Penicillin and streptomycin were purchased from Pfizer Inc. (New York, NY). Recombinant human tumour necrosis factor (rTNF) and recombinant human lymphotoxins (rLT) were the gifts of Genentech (South San Francisco, Calif.), $^3$H-thymidine was purchased from ICN (Irvine, Calif.) and mitomycin C was obtained from Sigma Inc. (St Louis, MO).

Polyclonal antisera to tumour necrosis factor and lymphotoxin were provided by Dr Gale Granger, University of Calif. Irvine and prepared as previously reported (Lewis *et al.*, 1977).

### *Separation and mitogenic stimulation of peripheral blood mononuclear cells (PBMC)*

Human peripheral blood mononuclear cells (PBMC) were obtained as the buffy coat fraction from normal human donors. PBMC were separated on Ficoll Hypaque gradients (Boyum, 1968), (specific gravity of 1.077) washed and adjusted to a final concentration of $2.5 \times 10^6$ cells ml$^{-1}$ in RPMI 1640 supplemented with 10% foetal bovine serum, 250 units of penicillin and 150 μg ml$^{-1}$ of streptomycin.

PBMC were stimulated with either 2.5 or 25 μg ml$^{-1}$ of PHA or 25 μg ml$^{-1}$ of Con A. Incubation of the cells was then carried out in 15 ml plastic centrifuge tubes in a 5% $CO_2$ incubator at 37°C for 3 days. At the end of that time, the cells were removed by centrifugation, tested for viability using trypan blue and exposed to $^3$H-thymidine as described below. Biological assays with these cells were performed only if the viability of the cells was 95% or greater. All supernatants harvested from the PBMC after stimulation were dialysed for 48 h against phosphate buffered saline with at least five bath changes and frozen for subsequent cytotoxin assay. The dialysis membrane used had a pore size that excluded molecules greater than 5,000 Daltons and the volume of the bath was 20 times greater than the volume of the samples being dialysed. Dialysis was performed to remove SMS. In those experiments where SMS was added to the PBMC to study the effects on production of cytotoxin, final dilutions of SMS were made just prior to addition to the PBMC. Appropriate controls were performed for each

Correspondence: T. Cesario.
Received 10 October 1990; and in revised form 9 April 1991.

test sample. Controls were simultaneously prepared and treated in every way identically to the test samples save for the absence of the SMS reagent.

*Proliferative responses*

Proliferative responses were determined by pulsing stimulated and unstimulated PBMC at a cell concentration of $1 \times 10^6$ cells $ml^{-1}$ for 4 h in a $CO_2$ incubator with $^3H$-thymidine (0.5 µCi/well) having a specific activity of 6.7 Ci $nM^{-1}$ (ICN, Irvine, CA). Aftr pulsing, the cells were harvested using a multisample automatic cell harvester. The samples were deposited on Filtermats (Skatron Inc., Lier, Norway) and the precipitates washed extensively. The $^3H$-thymidine incorporation was measured using a liquid scintillation counter.

*Cytotoxin assays*

Cytotoxin was assayed according to the methods reported previously (Yamamoto *et al.*, 1986). Briefly, confluent monolayers of mitomycin C treated murine L929 cells in 96 well flat bottom microtiter plates were exposed to serial 2-fold dilutions of sample. After overnight incubation, plates were stained with crystal violet, washed thoroughly with tap water and dried completely. The bound dye was eluted by 95% ethanol and measured spectrophotometrically in a Titertek Multiscan (Flow Laboratories, McLean, Va.) plate reader. The cytotoxin titers were estimated by plotting the absorbance as a function of the cytotoxin dilution and calculating the end point as that last dilution where 50% of the cells lysed (a 50% reduction in dye uptake). A standard cytotoxin preparation provided by Dr Granger was employed in every assay. The results are expressed in units $ml^{-1}$. A unit is the least amount of cytotoxin causing 50% destruction of the target cells.

In those experiments where the effect of SMS on bioactivity of rLT or rTNF was being tested, freshly diluted SMS in the appropriate concentration ($10^{-6}$ to $10^{-10}$ M) was added with a microdropper to individual wells of the plate. Immediately thereafter samples containing rLT or rTNF were titrated on the plates. The assay was then carried out as stated above. The titer of the cytotoxin as determined in the presence of the SMS was compared to the titer in the absence of the SMS.

*Typing of the cytotoxins*

Typing was accomplished by exposure of the L929 cells to antisera specific to either human rTNF or human rLT. Briefly, confluent monolayers of the L929 cells as above were exposed 10 µl of polyclonal rabbit antiserum made to either rTNF or rLT or, were exposed to an identical volume of normal rabbit serum. Antisera were adjusted to neutralise at least 200 units of activity prior to incubation and were shown to be specific for either rLT or rTNF without cross neutralisation at the dilutions used. After addition of the specific antiserum or normal control serum, supernatants prepared from the PBMC and shown to contain cytotoxins were added to the wells and the cytotoxin assay as described above completed. The per cent neutralisation (Lewis *et al.*, 1977) was calculated as

$$\frac{(1 \text{ -Titer in antiserum})}{\text{Titer in normal serum}} \times 100$$

*Statistical analysis*

Statistical analysis was performed using the Student's paired *t*-test.

### Results

In order to understand the effect of a compound on a biological substance it is necessary to examine the effects of

**Figure 1** The effect of SMs on the bioactivity of recombinant lymphotoxin (LT) in L929 cells. ☐ rLT without SMS, ▩ rLT with $10^{-7}$ M SMS, ▨ rLT with $10^{-8}$ SMS, ▧ rLT with $10^{-9}$ SMS, ■ rLT with $10^{-10}$ SMS. The increase in titer of the LT in the presence of SMS 28 at the $10^{-8}$ and $10^{-9}$ M concentration is significantly greater than the titer in the absence of SMS ($P < .05$). Similarly the increase in the titer of LT in the presence of SMS 14 at a concentration of $10^{-9}$ M is significant ($P < .01$). Each bar represents a mean of 12 experiments.

**Figure 2** The effect of SMS on the bioactivity of recombinant tumour necrosis factor. ☐ recombinant rTNF without SMS, ▩ recombinant TNF with SMS $10^{-7}$ M. ▨ rTNF with $10^{-8}$ M SMS, ▧ rTNF with $10^{-9}$ M SMS, ■ rTNF with $10^{-10}$ M SMS.

The increase in titer of TNF in the presence of $10^{-9}$ M SMS 28 is significant ($P < .05$). Each bar is derived from a mean of twelve experiments. The error bar for SMS 28 at the $10^{-8}$ M concentration ends exactly on the line at the edge of the figure.

the compound on both the production and the bioactivity of the substance. Thus to understand the effects of SMS we investigated the influence of SMS on the cytotoxicity of both rLT and rTNF. Figure 1 demonstrates the effect of the three forms of SMS on the bioactivity of recombinant lymphotoxin (LT). It can be seen that the 28 amino acid form of SMS (SMS 28) significantly enhanced the bioactivity of LT. The greatest increase was seen at the $10^{-8}$ M concentration where the presence of the peptide enhanced LT activity 4-fold. At this concentration enhanced activity was seen in 11 of the 12 experiments. At $10^{-9}$ M, SMS still enhanced activity. In addition, two forms of the 14 amino acid SMS moiety were tested. These represent the naturally occurring tetradecapeptide and the first 14 amino acids from the amino terminal of the 28 amino acid SMS moiety. The latter was included as a control. The natural 14 amino acid form of SMS 14 was shown (Figure 1) to enhance LT activity by a factor of four with peak activity at the $10^{-9}$ M concentration. This observed increase in activity was again seen in all of the 12 experiments. In contrast, SMS 28 (AA 1-14) had no effect on LT activity.

Control experiments with SMS of all three types demonstrated that the agent itself had no effect on the L929 cells

(when exposed to the cells for the standard 24 h incubation period of the assay) as demonstrated either by light microscopy or by the ability of the cells to stain with crystal violet. Thus the SMS was not toxic to the cells.

In Figure 2 we have illustrated the results of SMS on the bioactivity of TNF. It can be seen that SMS 28 enhanced TNF activity with significance achieved at the $10^{-9}$ M concentration. This increase in activity was seen in 11 of the 12 experiments run. Neither SMS 14 or SMS 28 (AA 1-14) affected the titer of the TNF to a statistically significant degree.

In Figure 3, we have shown the effect of SMS on the production of cytotoxins by PBMC. When PBMC are treated with SMS alone, no cytokines were detected in the supernatants from the treated PBMC. When similar cells were treated for 24 h with SMS 14, incubated for 24 h and then washed free of this tetradecapeptide and subsequently stimulated with 25 $\mu$g ml$^{-1}$ of PHA for 72 h, the titer of cytotoxins detected in the supernatant was the same as in supernatants taken from control PBMC induced with PHA but not exposed to SMS 14. Thus preincubation with removal of the SMS did not affect the ability of the cells to produce cytotoxin (data not shown). In contrast when SMS was added with the inducer (Figure 3), an adverse effect was appreciated on the titer of these cytotoxins induced in the presence of SMS as compared to the titer induced by PHA in the absence of SMS. Using the naturally occurring 14 amino acid moiety, a significant reduction in cytotoxin activity as compared to controls was appreciated at the $10^{-7}$ M and $10^{-8}$ M SMS concentrations. At both higher and lower dilutions of the SMS no effects were appreciated. Similar results were obtained when Con A was used as the inducer. In

**Figure 3** The effect of SMS 14 on the production of cytotoxins induced in PBMC. The first two bars indicate no cytotoxin was induced in the cells alone or in the presence of SMS 14. ☐ cytotoxin produced by PHA with $10^{-6}$ M SMS. ▩ cytotoxin produced by PHA + $10^{-7}$ M SMS, ▨ cytotoxin produced by PHA + $10^{-8}$ M SMS. ▧ cytotoxin produced by PHA with $10^{-9}$ M SMS. ■ Cytotoxin produced by PHA with $10^{-10}$ M SMS. PHA alone ⊠. The reduction in titer in the presence of $10^{-7}$ M and $10^{-8}$ M SMS is significant ($P < .05$). Each bar is derived from a mean of 24 experiments performed on cells form 12 separate donors.

virtually every test run with the naturally occurring form of SMS, the yield of cytotoxin was reduced. The 28 amino acid moiety had no effects on production of these cytokines (data not shown) including under those circumstances when the PHA concentration was reduced to suboptimal levels (i.e., 0.25 and 2.5 $\mu$gm ml$^{-1}$).

When the cytotoxin produced by PBMC was neutralised using specific antisera, we found the cytotoxic activity induced by PHA was neutralised by antitoxin specific for both TNF and LT. The same was true of the cytotoxin induced by PHA in the presence of $10^{-8}$ M SMS (Table I).

In contrast to previous studies (Stanisz *et al.*, 1986; Wagner *et al.*, 1979; Payan *et al.*, 1984; Mascardo, 1984) we could not demonstrate a reduction in the proliferative responses over the concentrations demonstrated here.

### Discussion

Somatostatin, a neuropeptide, has been reported to inhibit T cell proliferation and influence other immune functions (Stanisz *et al.*, 1986; Wagner *et al.*, 1979; Payan *et al.*, 1984; Mascardo *et al.*, 1984; Yousefi, *et al.*, 1990). In the past, we have reported that SMS of both 14 and 28 amino acid species will decrease the production of human interferon gamma but not interferon alpha (Yousefi *et al.*, 1990). In this report we have demonstrated the paradoxical effects of SMS on cytotoxins produced by the immune system.

It is of interest that recombinant SMS in both 14 and 28 amino acid forms enhanced activity of LT on L929 cells. This approximately 3-fold enhancement occurred at concentrations slightly above those encountered physiologically (Labhart, 1986) but could approximate the ranges of concentrations encountered in the therapy of neoplastic diseases with SMS analogs (Kutz *et al.*, 1986). It is of interest that cytotoxic activity is statistically significantly increased over a very narrow range of concentrations not including the highest concentrations tested. This same phenomena has been observed elsewhere (Payan *et al.*, 1984; Pawlikowski *et al.*, 1985).

In our data, enhancement of bioactivity by naturally occurring forms of SMS was consistently observed with both LT and TNF at the $10^{-9}$ M concentrations except when studying SMS 14 and TNF. SMS in all forms tested was not toxic to the cells in the absence of cytotoxins. We suggest SMS could promote injury caused by the cytotoxins by inhibiting the synthesis of enzymes used to repair damage induced by LT and TNF.

It is not surprising that SMS 28 (AA 1-14) had no effect on LT activity. The last 14 amino acid fragment of SMS 28, i.e. the carboxyl terminal, bears strong resemblance to the naturally occurring 14 amino acid moiety. The last 11 amino acids from the carboxyl terminal are identical between the two molecules and may represent the active component in its effects on the immune system. Similar effects were seen on gamma interferon production (Yousefi *et al.*, 1990).

SMS 28 also significantly enhanced TNF activity at the $10^{-9}$ concentration. The 14 amino acid form did increase activity, but not to a statistically significant degree. That differences exist in the effect of LT and TNF in the presence of SMS is not surprising because recent information has

**Table I** Typing of the peripheral blood mononuclear cell derived cytotoxin using specific antisera

| *Treatment* | *Titer before treatment** | *Percent neutralisation with anti-LT serum* | *Percent neutralisation with anti-TNF serum* | *Percent neutralisation with both antisera* |
|---|---|---|---|---|
| Recombinant LT | 100 ± 23 | 100 | 0 | – |
| Recombinant TNF | 205 ± 15 | 0 | 100 | – |
| PBMC + PHA | 557 ± 123 | 80 | 72 | 100 |
| PBMC + PHA + SMS ($10^{-8}$ M) | 183 ± 27 | 67 | 70 | 100 |

*Mean of three experiments.

suggested that the effects of these two cytokines may differ in various cell types (Browning & Ribolini, 1989).

Since SMS does inhibit the secretion of other peptides (Brazeau et al., 1973; McCann et al., 1980) including those of the immune system (Yousefi et al., 1990), it might be anticipated that it would also decrease the secretion of cytotoxic cytokines. This inhibition occurs in ranges that are above those encountered in the circulation (Labhart, 1986). However, such levels of SMS could possibly be found in areas proximate to the cells secreting SMS (paracrine effect). Further, as cells of the immune system have been reported to produce SMS (Goetzl et al., 1985), an autocrine effect might be possible.

Results presented here suggest that SMS 14 is responsible for inhibition of the production of cytotoxic cytokines but that SMS 28 is not. This is again in keeping with the differing effects of these SMS moieties (Gordon et al., 1989). Our data suggest that SMS affected both TNF and LT synthesis since neutralisation experiments with specific antisera showed cytotoxin produced in the presence of SMS was partially neutralised by both anti-TNF and anti-LT sera. This is identical to results on the cytotoxin produced by PHA without SMS. The production of TNF and LT are regulated differently (Centuri et al., 1987), and these two cytokines are produced by dissimilar cell types (Goeddel et al., 1986; Paul & Ruddle, 1988), thus it is possible an agent could affect the production of one cytotoxin, but not the other. The mechanism of the inhibition in production of cytokines could possibly relate to the fact SMS may influence membrane permeability to calcium (Pace & Tarvin, 1981) which is important in the secretion of other cytokines (Cesario et al., 1988). Since SMS does affect bioactivity, we had exhaustively dialysed (with five bath changes) supernatants to remove SMS. Furthermore a pore size was selected in the dialysis membrane that easily allows SMS to pass, but does not affect passage of the cytokines.

We did not find an effect of SMS on proliferation in contrast to the work of others (Payan et al., 1984; Mascardo et al., 1984; Pawlikowski et al., 1985). This is our case may relate to the shorter (4 h) exposure period for thymidine uptake we have used for convenience since our major thrust is cytokine production. We have reported in earlier studies that proliferation was decreased using octreotide (Yousefi et al., 1990); however, previous work with gamma interferon has established that secretion of cytokines can be dissociated from the proliferative response (Berenbaum et al., 1975).

Our findings may have some clinical relevance. The significant enhancement of LT and TNF found here *in vitro* with murine cells if encountered in the body and against other cell types suggests SMS could influence tumour growth by potentiating the cytotoxic effects of LT and TNF as well. This hypothesis will remain for on-going investigations to verify.

### References

BERENBAUM, M., FLUCK, P. & HURST, N. (1975). Depression of lymphocyte responses after surgical trauma. *Br. J. Exp. Path.*, **54**, 597.

BOYUM, A. (1968). Isolation of mononuclear cells and granulocytes from human blood. *Scand. Journ. Clin. Invest.*, **97**, (Suppl.) 77.

BRAZEAU, P., VALE, W. & BURGUS, R. (1973). Hypothalamic peptide that inhibits the secretion of immunoreactive pituitary growth hormone. *Science*, **179**, 77.

BROWNING, J. & RIBOLINI, A. (1989). Studies on the differing effects of tumor necrosis factor and lymphotoxin on the growth of several human tumor lines. *J. Immunol.*, **143**, 1859.

CERTURI, M., MURPHY, M., COSTA-GROMI, M., WEINMANN, R., PERUSSIA, B. & TRINCHIERI, G. (1987). Independent regulation of tumor necrosis factor and lymphotoxin production by human peripheral blood lymphocytes. *J. Exp. Med.*, **165**, 1581.

CESARIO, T., MCCLOSKEY, M., CARANDANG, G., YOUSEFI, S., CHIU, J. & VAZIRI, N. (1988). Calcium and the production of interferon by human peripheral blood mononuclear cells. *J. Interferon Res.*, **8**, 783.

GOEDDEL, D., AGGARWAL, B., GRAY, P. & 6 others (1986). Tumor necrosis factor: gene structure and biological activities. Cold Spring Harbor Symposium in *Quant. Biol.*, **51**, 597.

GOETZL, E., CHERNOV-ROGAN, T., COOKE, M., RENOLD, F. & PAYAN, D. (1985). Endogenous somatostatin-like peptides of rat basophilic leukemia cells. *J. Immunol.*, **135**, 2707.

GORDON, P., COMI, R., MATON, P. & GO, V. (1989). Somatostatin and somatostatin analogues (SMS201-955) in the treatment of hormone secreting tumors of the pituitary and gastrointestinal tract and non-neoplastic diseases of the gut. *Ann. Internal Med.*, **110**, 35.

GUSTAVSSON, S. & LUNDQUIST, G. (1978). Inhibition of pancreatic somatostatin release in response to glucose. *Biochem. Biophys. Res. Commun.*, **82**, 1229.,

KUTZ, K., NUESCH, E. & ROZENTHALER, J. (1986). Pharmacokinetics of SMS 201-995 in healthy subjects. *Scand. J. Gastroenterol.*, **119**, (Suppl. 21) 65.

LABHART, A. (1986). Tissue Hormones. In *Clinical Endocrinology*. (Second Edition) Labhart, A. (ed.). Springer-Verlag: New York.

LEWIS, J., CARMACK, C., YAMAMOTO, R. & GRANGER, G. (1977). Antibodies against human lymphokines. I. Methods for induction of antibodies capable of neutralizing stable alpha and unstable beta lymphotoxins released in vitro by activated lymphocytes. *J. Immuno. Methods*, **14**, 163.

MASCARDO, R., BARTON, R. & SHARLINE, P. (1984). Somatostatin has an antiproliferative effect on concanavalin A activated rat thymocytes. *Clin. Immunol. & Immunopath.*, **33**, 131.

MCCANN, S., KURLICH, L., NEGRO-VILAR, A., OJEDA, S. & VIJAYAN, E. (1980). Regulation and function of panhibin (Somatostatin). *Adv. Biochem. Psychopharmacol.*, **2**, 131.

PACE, C.S. & TARVIN, J.T. (1981). Somatostatin: mechanism of action on pancreatic islet cells. *Diabetes*, **30**, 836.

PAUL, N. & RUDDLE, N. (1988). Lymphotoxin. *Ann. Rev. Immunol.*, **6**, 407.

PAWLIKOWSKI, M., STEPIEN, H., KUNERT-RADEK, J. & SHALLY, A. (1985). Effect of somatostatin on the proliferation of mouse spleen cells *in vitro*. *Biochem. Biophys. Res. Commun.*, **129**, 52.

PAYAN, D., HESS, C. & GOETZL, E. (1984). Inhibition by somatostatin of the proliferation of T lymphocytes and Molt 4 lymphoblasts. *Cellular Immunol.*, **84**, 433.

REICHLIN, S. (1983). Somatostatin. *N. Eng. J. Med.*, **309**, 1495.

SCHALLY, A. (1988). Oncological applications of somatostatin analogs. *Cancer Res.*, **48**, 6977.

SCHALLY, A., HUANG, W., CHANG, P. & 5 others (1980). Isolation and structure of pre somatostatin: a putative somatostatin precursor from pig hypothalamus. *Proc. Natl Acad. Sci. USA*, **77**, 4489.

STANISZ, A., BEFUS, D. & BIENENSTOCK, J. (1986). Differential effects of vasoactive intestinal peptide, substance P and somatostatin on immunoglobulin synthesis and proliferation by lymphocytes from Peyer's patches, mesenteric lymph nodes and spleen. *J. Immunol.*, **135**, 152.

WAGNER, M., MENGST, K., ZIERDEN, E. & GERLACH, U. (1979). Investigations of the antiproliferative effect of somatostatin in man and rats. *Metab. Clin. Exp.*, **27**, 1381.

WUNSCH, E., JAEGER, F., MORODER, L., PEGGIM, E. & PALUMBO, M. (1981). Somatostatin-28: a conformational analysis. *Biopolymers*, **20**, 1741.

YAMAMOTO, R., WARE, C. & GRANGER, G. (1986). The human LT system. XI Identification of LT and TNF like LT forms from stimulated natural killers, specific and nonspecific cytotoxic human T cells in vitro. *J. Immunol.*, **137**, 1978.

YOUSEFI, S., GHAZINOURI, A., VAZIRI, N., TILLES, J., CARDANDANG, G. & CESARIO, T. (1990). The effect of somatostatin on the production of human interferons by mononuclear cells. *Proc. Soc. Exp. Biol. Med.*, **194**, 114.